# Research on industrial carbon emission prediction and resistance analysis based on CEI-EGM-RM method: a case study of Bengbu

**DOI:** 10.1038/s41598-023-41857-0

**Published:** 2023-09-04

**Authors:** Dawei Dai, Biao Zhou, Shuhang Zhao, Kexin Li, Yuewen Liu

**Affiliations:** 1 State Key Laboratory of Mining Response and Disaster Prevention and Control in Deep Coal Mines, Anhui University of Science and Technology, Huainan, Anhui China; 2 School of Humanities and Social Sciences, Anhui University of Science and Technology, Huainan, Anhui China; 3 School of Economics and Management, Anhui University of Science and Technology, Huainan, Anhui China

**Keywords:** Ecology, Environmental sciences, Environmental social sciences

## Abstract

This paper focuses on the development trend of industrial carbon emissions in Bengbu city, Anhui Province in the next ten years, and how to help the industry reach the carbon peak as soon as possible. The research process and conclusions are as follows: (1) Through literature review and carbon emission index method, five main factors affecting industrial carbon emission are identified. (2) The resistance model is used to analyze the main resistance factors of industrial carbon emission reduction in Bengbu city. (3) Based on the existing data of Bengbu city from 2011 to 2020, the grey prediction EGM (1,1) model is used to predict the industrial carbon emissions of Bengbu city from 2021 to 2030. The results show that among the five factors, the urbanization rate has the most significant impact on industrial carbon emissions, while energy intensity has the least impact. Bengbu’s industrial carbon emissions will continue to increase in the next decade, but the growth rate will be flat. Based on the findings of the analysis, specific recommendations on urbanization development, energy structure, and industrial structure of Bengbu city are put forward.

## Introduction

Since the industrial revolution, human activities, especially the industrialization process of developed countries, have consumed a large amount of fossil energy, leading to a rapid increase in greenhouse gas emissions. Global warming is one of the biggest challenges facing the world in the twenty-first century. To cope with climate change and promote the building of a community with a shared future for humanity, all countries need to reduce greenhouse gas emissions jointly. According to the World Meteorological Organization (WMO), the Earth is now nearly one degree Celsius warmer than before industrialization began. On this trend, global temperatures will be 3 to 5 °C above pre-industrial levels by 2100^1^. If no action is taken, climate change will have a severe impact on economic and social development at current trends. Moreover, climate issues have large-scale spatial and temporal externalities that require a coordinated global response.

China is the world’s largest industrial country and carbon emitter^2, 3^. Since the reform and opening up, China’s economic and social development has achieved remarkable results, but it has also led to the continuous growth of carbon emissions, causing significant damage to the natural environment. On September 22, 2020, the Chinese government solemnly pledged at the 75th session of the United Nations General Assembly that China would increase its intended nationally determined contributions (INDC), strive to peak its carbon dioxide emissions by 2030, and achieve carbon neutrality by 2060. Cities are essential parts of China’s carbon emissions, the main carrying space of carbon sink function, and the critical administrative units for implementing of dual carbon goals and policies. In November 2021, the National Development and Reform Commission (NDRC) issued the implementation plan for high-quality development of industrial transformation and upgrading demonstration zones in old industrial cities and resource-based cities during the 14th Five-Year Plan Period to support cities in promoting industrial restructuring and green and low-carbon transition, leading the revitalization and development of old industrial cities and resource-based cities nationwide^4^. On October 16, 2022, the report of the 20th National Congress of the Communist Party of China emphasized again the promotion of green development, and actively yet prudently promoting carbon peaking and carbon neutrality^5^. So, the transformation and development of industrial cities is imperative.

As the sector with the most significant CO_2_ emission, which factors will affect industrial carbon emission intensity is a scientific proposition that must be understood to achieve structural emission reduction. In recent years, especially since the Chinese government put forward the goal of peaking carbon emissions by 2030, many experts and scholars in the academic circle have conducted much research on carbon emissions and peak prediction of industrial industries. The main focus is on the following two aspects: identification of factors affecting industrial carbon emissions and prediction of carbon peaks.

The identification of factors influencing industrial carbon emissions is a focus of academic concern. In this regard, domestic and foreign scholars have conducted a large number of studies on the factors affecting CO_2_ emissions, and found that they are mainly affected by economic growth^6–8^, energy efficiency^6, 9^, carbon emission intensity^10, 11^, industrial structure^8, 12^, and urbanization rate^8, 13^. In the prediction of industrial carbon peaking, standard research methods include gray prediction^14^, scenario analysis method^15^, and BP neural network^16^. The research perspective is mainly from the national overall macro perspective^15, 17^, provincial perspective^18, 19^, and regional^20, 21^. Meanwhile, most research targets focus on resource-based cities^22–24^ or low-carbon pilot cities^25–27^.

In summary, it is not difficult to find many studies on industrial carbon emissions and carbon peaking in existing literature, and relatively affluent research results have been achieved. However, there are still the following shortcomings. On the one hand, in constructing of the evaluation index system of influencing factors of industrial carbon emissions, few scholars have comprehensively evaluated it from four dimensions: population, structure, economy, and technology. On the other hand, the realization of the national energy conservation and emission reduction strategy goals cannot be achieved without the achievement and support of the carbon emission indicators of each city. Unfortunately, the current research on carbon emissions is mainly from the macro perspective of China’s overall region or the perspective of provinces, with insufficient attention paid to small and medium-sized cities, especially industrial cities.

Therefore, to make up for this deficiency, this paper selects Bengbu city, an essential comprehensive industrial base in Anhui Province, as the research object. Based on previous research, a thorough evaluation index system for industrial carbon emissions is constructed from four aspects: population, structure, economy, and technology. Combining the carbon emission index (CEI) method and resistance model to thoroughly analyze the influencing factors and resistance factors of carbon emission reduction, the EGM(1,1) model and linear regression equation model are applied to predict the future carbon emission trend accurately. Furthermore, the analysis results are used to explore the realization path of carbon emission reduction in Bengbu city.

Bengbu city is an essential old industrial base in Anhui Province with a relatively complete industrial system focusing on light textile, heavy industry and chemical industry. At the same time, it is also a provincial leading demonstration city of energy efficiency. In recent years, influenced by factors such as slow industrial transformation and upgrading and green development strategy, the economic growth of Bengbu city has stalled. In 2020, the industrial GDP increased by 0.03% compared to the previous year, but the industrial carbon emission remained at a relatively high level (See the Results section for detailed calculations). Therefore, it is vital for Bengbu city to coordinate the relationship between economic development and carbon emission by studying the current situation and future trends of industrial carbon emission in Bengbu city and exploring the path of green transformation for Bengbu city. In view of this, we study its carbon emission resistance factor and carbon emission trend. Since it will be less than ten years for China to reach the carbon emission peak, this paper focuses on predicting whether the industry can achieve the carbon emission peak before 2030, and regulating which factors can promote the smooth realization of the target. Finally, according to the analysis results, relevant targeted recommendations are provided for Bengbu’s carbon emission reduction, and reference for other similar industrial cities’ carbon emission reduction actions.

## Materials and methods

### Research area

**Figure 1:**
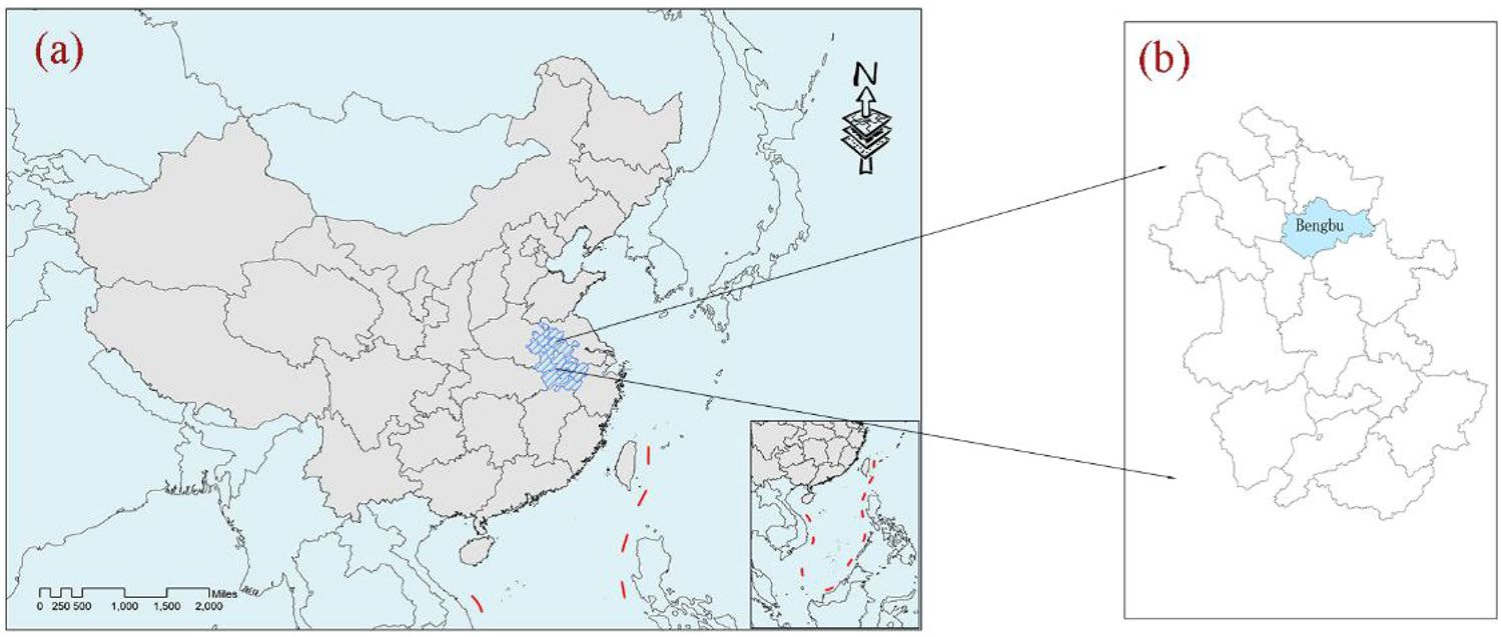
Location map of Anhui Province and Bengbu city in China. **(a)** Location map of Anhui Province in China. **(b)** Location map of Bengbu city in Anhui Province.

Bengbu city is located east of China, west of the Yangtze River Delta, and north of Anhui Province (Fig. 1), with a total area of 5,951 square kilometers. It is an important comprehensive industrial base in Anhui Province with a relatively complete industrial system focusing on light textile, heavy industry, and chemical industry. In 2020, the city’s permanent population was 3.3 million, with an urbanization rate of 41%. There are 1,061 industrial enterprises above designated size, with a total industrial output value of 67.46 billion yuan, accounting for 32% of Bengbu’s GDP. Meanwhile, industry accounts for nearly 50% of total carbon emissions. In conclusion, industry has a very important role in effectively achieving carbon emission reduction in Bengbu city, which needs further analysis.

### Data sources

The data in this paper mainly come from Bengbu Statistical Yearbook, and the carbon emissions are calculated using the carbon emissions measurement method in IPCC National Greenhouse Gas Inventory Guide. The data of Bengbu city from 2011 to 2020 are selected as the basic research data, and other relevant data are calculated by CEI method and resistance model, predicted by grey prediction model and linear regression equation model.

### Calculation method of industrial carbon emissions

The primary source of carbon dioxide emission is the combustion of fossil energy. China’s energy carbon dioxide emission occupies an absolute proportion of total carbon emission. Therefore, the carbon emission of Bengbu city measured in this paper specifically refer to the carbon emission generated by the combustion of fossil energy.

According to the general method of IPCC guidelines, this paper calculates the carbon emissions generated when fossil energy is burned, as Eq. (1).

1 $${C}_{i}=\sum {B}_{j}{c}_{j}$$

${C}_{i}$ is the carbon emissions of industry *I* (ten thousand tons); ${B}_{j}$ is the energy consumption after discounting standard coal; ${c}_{j}$ is the carbon emission factor; $j$ is the type of energy.

The coal coefficient and carbon emission coefficient of each energy are shown in Table 1.

**Table 1:** Fold standard coal coefficient and carbon emission coefficient of main energy.

Energy	Fold standard coal coefficient	Carbon emission factor
Coal	0.7146	0.7550
Coke	0.9714	0.8550
Kerosene	1.4714	0.5714
Diesel	1.4571	0.5921
Gasoline	1.4714	0.5538
Fuel–oil	1.4286	0.6185
Natural gas	1.3300	2.1622
Liquefied petroleum gas	1.7143	0.5042

### CEI method

The carbon emission index method is used to determine the weight of indicators, which can avoid subjective factors in the weighting^28^. The specific calculation steps are as follows:

Step 1: Data standardization. Calculated by Eqs. (2) and (3).

2 $${\mathrm{Positive \, indicators}: X}_{ij}=\frac{{a}_{ij}-min\{{a}_{ij}\}}{\mathit{max}\left\{{a}_{ij}\right\}-min\{{a}_{ij}\}} (i=\mathrm{1,2},\cdots ,\mathrm{m};j=\mathrm{1,2},\cdots ,\mathrm{n})$$

3 $$\mathrm{Negative\, indicators}: {X}_{ij}=\frac{\mathit{max}\left\{aij\right\}-aij}{\mathit{max}\left\{aij\right\}-min\{aij\}}(i=\mathrm{1,2},\cdots ,m;j=\mathrm{1,2},\cdots ,n)$$

where, *aij* is the *JTH* index of the *ITH* year.

Step 2: Calculate the proportion of item* j* in the *ITH* year, namely Eq. (4).

4 $${P}_{ij}=\frac{{X}_{ij}}{\sum_{i=1}^{m}{X}_{ij}}$$

Step 3: Calculate the entropy value of the *JTH* index. The formula is shown in Eq. (5).

5 $${e}_{j}=-\frac{1}{lnm}{\sum }_{i=1}^{m} ({P}_{ij}ln{P}_{ij}) {e}_{j}\epsilon [0, 1]$$

Step 4: Calculate the coefficient of difference. The formula is as Eq. (6).

6 $${g}_{i}=1-{e}_{j}$$

Step 5: Calculate the weight of the *ITH* index, and the formula is Eq. (7).

7 $${W}_{i}=\frac{gi}{{\sum }_{j=1}^{n}gi}$$

### Resistance model

In analyzing of influencing factors of industrial carbon emission, it is more necessary to analyze and diagnose the critical resistance factors in carbon emission problem, to provide specific carbon emission reduction recommendations for the region. Therefore, this paper introduces a resistance model to study the influencing factors of industrial carbon emissions in Bengbu city, and further explore the main resistance factors affecting the peak of industrial carbon emissions.

The resistance model is calculated using three indexes: factor contribution degree, index deviation degree and resistance value^29^. The calculation formula is Eq. (8).

8 $${O}_{i}=\frac{{S}_{i}{W}_{i}}{\sum_{i=1}^{n}({S}_{i}{W}_{i})}$$

Among them, the resistance value ${O}_{i}$ represents the influence degree of each index; index deviation ${S}_{i}=1-{X}_{ij}$, which means the difference between each index and the optimal value.

### Grey prediction model EGM (1,1)

In 1982, to effectively solve the problem of small data, uncertainty system analysis and prediction, Professor Deng put forward the grey system theory, which has been widely used in many fields. Grey system theory is mainly based on the known part of the information to study and extract, correctly describe the law of system evolution, to predict future changes. At present, grey system theory has been successfully applied to many field and can solve problems in particular areas with unknown factors. It takes the uncertain system with some information known, some information unknown, small samples, and poor information as the research object. Meanwhile, it can realize the accurate description of the system operation behavior and evolution law, has the advantages of simple operation, high precision and easy to test, and is often used for energy index prediction^30^. Grey prediction model EGM(1,1) is one of the most widely used grey system models and has a good prediction effect for data samples with fewer years. This paper uses ten years of data on industrial carbon emissions in Bengbu city to make predictions, so the prediction results is effective using this method. 

The construction method of EGM (1,1) is as follows:

Step 1: Assume that the original data series is Eq. (9).

9 $${X}^{(0)}={(x}^{\left(0\right)}\left(1\right),{x}^{\left(0\right)}\left(2\right),\cdots ,{x}^{\left(0\right)}\left(n\right))$$

Using a single additive generation operation, Sequence ${X}^{(0)}$ can be generated into a new sequence ${X}^{(1)}$ (shown in Eq. 10).

10 $${X}^{\left(1\right)}=\left\{{x}^{\left(1\right)}\left(1\right),{x}^{\left(1\right)}\left(2\right),\cdots ,{x}^{\left(1\right)}\left(n\right)\right\}= \left\{{\sum }_{m=1}^{1}{x}^{\left(0\right)}\left(m\right) {\sum }_{m=1}^{2}{x}^{\left(0\right)}\left(m\right) \cdots {\sum }_{m=1}^{n}{x}^{\left(0\right)}\left(m\right)\right\}$$

Step 2: Using the new sequence ${X}^{(1)}$ obtained, the general form of EGM (1,1) model is established, which is described by Eq. (11).

11 $$\frac{d{x}^{\left(1\right)}}{dt}+a{x}^{(1)}=b$$

*a* and *b* are correlation coefficients, which can be obtained by fitting the least square method.

Step 3: Build a grey prediction model: the solution of the differential equation can be obtained by Eq. (12).

12 $${\widehat{X}}^{\left(1\right)}\left(k\right){=({x}^{\left(1\right)}\left(0\right)-\frac{b}{a})e}^{-a\left(k-1\right)}+\frac{b}{a},k=\mathrm{1,2},\cdots ,n$$

Step 4: the reduced value of the original data is obtained by Eq. (13).

13 $${\widehat{X}}^{\left(0\right)}\left(k\right)={\widehat{X}}^{\left(1\right)}\left(k\right)-{\widehat{X}}^{\left(1\right)}\left(k-1\right),k=\mathrm{1,2},\cdots ,n$$

## Results

### Weight analysis of influencing factors

In recent years, many scholars have conducted relevant studies on the influencing factors of industrial carbon emissions. According to the actual situation of Bengbu city and based on previous studies, the availability of data is considered. Through literature review, industrial GDP, energy intensity, urbanization rate, proportion of secondary industry and carbon dioxide intensity were selected as indicators of industrial carbon emissions in Bengbu city from four aspects: population, structure, economy and technology (Table 2). And the descriptive statistics of each indicator are shown in Table 3.

**Table 2:** Identification of influencing factors of industrial carbon emissions.

Category	Index layer	Index attribute	References
Population	Urbanization rate (X1)	+	Sun and Huang^8^; Xiong et al.^31^
Structure	Proportion of secondary industry (X2)	−	Wen and Liu^32^; Liu and Liu^33^
Economy	Industrial GDP (X3)	+	Liu and Deng^34^; He and Wei^35^
Technology	Energy intensity (X4)	−	Tang et al.^36^; Ahmadi et al.^37^
	Carbon dioxide intensity (X5)	−	Guo et al.^38^; Liu et al.^39^

**Table 3:** Indicators description.

Indicators	Descriptions	Units
Urbanization rate	Urban population as a percentage of the total population	%
Proportion of secondary industry	Ratio of GDP of secondary industry to total GDP	%
Industrial GDP	Gross value of industrial output	Billion yuan
Energy intensity	Energy consumption per unit of GDP	Tons of standard coal/10,000 yuan
Carbon dioxide intensity	Carbon dioxide emissions per unit of GDP growth	Ton/10,000 yuan

Through the data collection from the Statistical Yearbook of Anhui Province and Bengbu Statistical Yearbook from 2011 to 2020, the relevant data are substituted into the calculation formula (1) of industrial carbon emissions to obtain the industrial carbon emissions of Bengbu city. Basic data are shown in Table 4. The weights of each index are obtained by standardizing and non-dimensionalizing the data of the five major evaluation indexes. The calculation results are shown in Table 5. According to the weight of each factor, the influencing factors of industrial carbon emissions in Bengbu city from large to small are urbanization rate, the proportion of secondary industry, industrial GDP, carbon dioxide intensity, and energy intensity.

**Table 4:** Identification of influencing factors of industrial carbon emissions.

Year	Coal	Coke	Kerosene	Diesel	Gasoline	Fuel–oil	NG	LPG	CE
2011	405.499	0.003	0.170	1.465	0.534	0.142	0.686	0.031	310.843
2012	389.721	0.061	0.161	1.425	0.624	0.094	0.796	0.015	301.051
2013	397.106	0.001	0.030	1.218	0.314	0.088	1.425	0.015	306.598
2014	394.850	0.000	0.001	1.330	0.227	0.086	1.789	0.070	305.365
2015	399.121	0.000	0.009	1.508	0.271	3.897	1.791	0.091	311.328
2016	287.773	0.018	0.001	1.628	0.285	6.279	2.055	0.077	229.051
2017	295.339	0.005	0.001	1.628	0.278	4.804	2.691	0.051	234.357
2018	424.596	0.125	0.007	1.052	0.254	3.224	2.136	0.048	330.752
2019	469.732	0.173	0.001	1.046	0.175	3.730	2.511	0.051	365.041
2020	437.772	0.426	0.000	1.453	0.100	3.384	2.926	0.040	340.910

Each energy unit is ten thousand tons of standard coal, carbon emission unit is ten thousand tons.NG means natural gas.

*LPG* liquefied petroleum gas, *CE* carbon emissions.

**Table 5:** The entropy value, difference coefficient and weight of the evaluation index.

	X1	X2	X3	X4	X5
The weight coefficient (${W}_{i}$)	0.327	0.211	0.160	0.149	0.153

### Analysis of resistance factors

**Figure 2:**
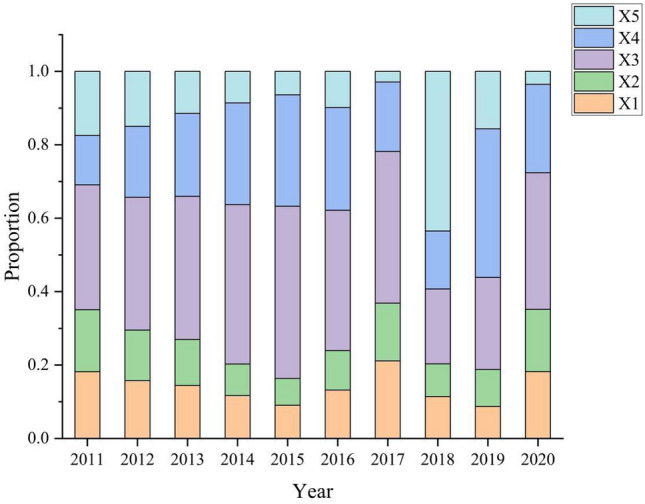
Chart of annual proportion of each index.

According to the analysis results of CEI method, the resistance model is introduced to analyze the resistance degree of each influencing factor. The resistance analysis of the influencing factors of industrial carbon emissions in Bengbu city from 2011 to 2020 is carried out, and ranked according to the resistance value. The calculation results are shown in Fig. 2. It can be discovered that the frequency of urbanization rate as the main resistance factor in the past ten years is eight, indicating that the accelerating urbanization process has a severe impact on the carbon emission reduction of Bengbu city, aggravating the increase of carbon emissions and bringing tremendous pressure to the environment. The relationship between industrial GDP and the proportion of secondary industry is relatively strong, indicating that as the economy develops quickly, industrial GDP is increasing and the share of secondary industry is relatively high. Nonetheless, the secondary industry consumes a lot more energy than the other two industries, which makes it difficult to reduce carbon emissions. Compared with the first three factors, energy intensity and carbon dioxide intensity have little influence, but they are still vital resistance factors, indicating that the energy use efficiency of Bengbu city needs to be improved.

### Analysis of grey prediction EGM (1,1)

To ensure the reliability of data prediction, this paper uses MATLAB R2017a software to simulate and predict the carbon emissions of Bengbu from 2011 to 2030. At the same time, the linear regression equation model is used to compare with this method, and the residual and relative error of the predicted and actual industrial carbon emissions of Bengbu from 2011 to 2020 are summarized as shown in Table 6. It is found that the relative error is similar to that of the linear regression prediction model. Then, using the data from 2011 to 2019 as the fitting data the grey prediction model and linear regression model are used to predict the carbon emissions in 2020, which are 340.91 and 344.24 million tons, respectively. So, no matter the linear regression model or the grey prediction model, the error between the calculated value and the actual value is very small. Meanwhile, the posterior difference test method was used to test the model’s progress. The calculation accuracy table of the prediction model is shown in Table 7. The mean square deviation *C* = 0.3143, *P* = 1, and the model accuracy is level 1, indicating a good level of simulation accuracy. In addition, the advantage of the grey prediction model is that it can forecast with time series as the independent variable. In this paper, the original data series of Bengbu city has been showing an upward trend. That is, it displays a certain rule, and the prediction can be made when the grey prediction model meets four data series.

**Table 6:** Table of test calculation and analysis of prediction model of industrial carbon emission in Bengbu city from 2011 to 2020.

Year	${X}^{(0)}$	EGM(1,1)			Linear regression prediction		
		${\widehat{X}}^{\left(0\right)}$	Residual error	Relative error	${\widehat{X{\prime}}}^{\left(0\right)}$	Residual error	Relative error
2011	317.07	317.07	0.89	0.00	311.71	5.36	0.02
2012	318.58	317.69	0.77	0.00	315.32	3.25	0.01
2013	320.56	321.33	1.93	0.00	318.94	1.62	0.01
2014	323.08	325.01	2.21	0.01	322.55	0.53	0.00
2015	326.52	328.73	1.05	0.01	326.17	0.36	0.00
2016	331.45	332.50	2.99	0.00	329.78	1.67	0.01
2017	339.30	336.31	6.32	0.01	333.40	5.91	0.02
2018	346.48	340.16	2.88	0.02	337.01	9.47	0.03
2019	346.94	344.06	7.09	0.01	340.62	6.32	0.02
2020	340.91	348.00	0.89	0.02	344.24	3.33	0.01

**Table 7:** Prediction model calculation accuracy standard grade table.

Model accuracy level	C	P
Good	C ≤ 0.35	0.95 ≤ P
Qualified	0.35 < C ≤ 0.5	0.80 ≤ P < 0.95
Barely qualified	0.5 < C ≤ 0.65	0.70 ≤ P < 0.80
Unqualified	0.65 < C	P < 0.70

**Figure 3:**
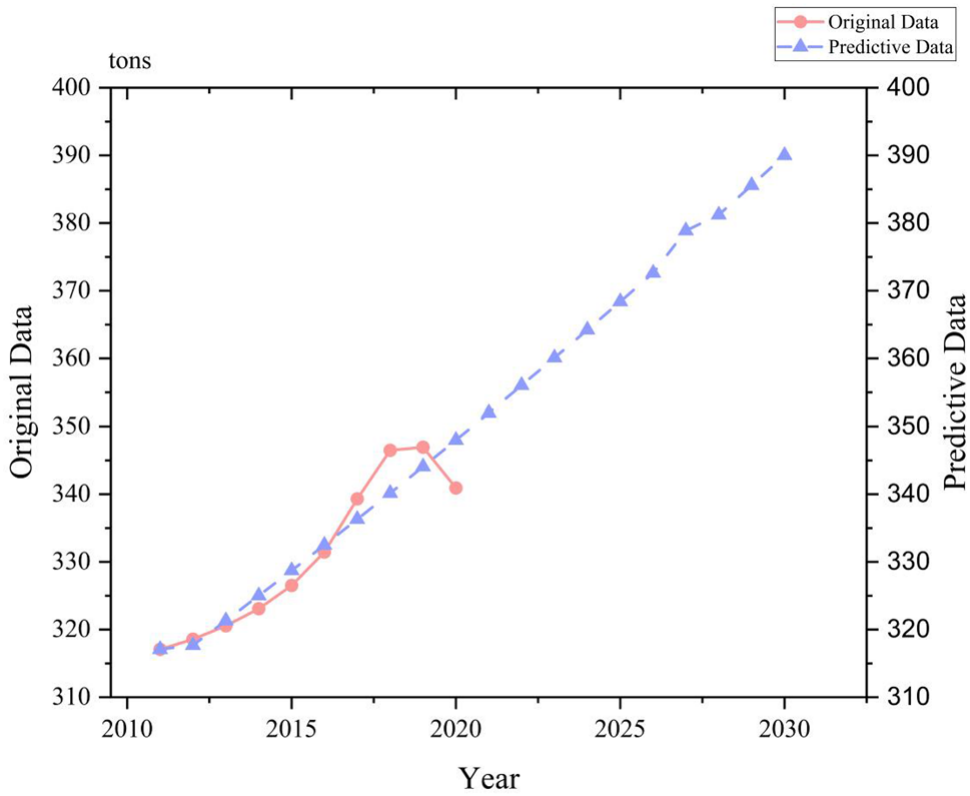
Grey prediction of industrial carbon emission in Bengbu city.

The results of EGM (1,1) simulation and prediction of industrial carbon emissions in Bengbu from 2011 to 2030 are shown in Fig. 3. It is worth noting that Bengbu’s industrial carbon emissions have been rising rapidly before 2019, and the growth rate began to slow down in 2019 and showed a downward trend in 2020. This indicates that the industrial transformation and green development strategy of Bengbu city have achieved certain results in recent years, and carbon emissions have been reduced.

## Discussion

According to the calculation results of CEI method and resistance model, urbanization rate has the most significant impact on industrial carbon dioxide emissions, the proportion of secondary industry has a greater effect on industrial carbon dioxide emissions, and energy intensity has a relatively small impact, which is consistent with the conclusion of references^8, 9, 12, 13^. This shows that urbanization construction is a severe problem facing Bengbu city. With the continuous expansion of urbanization scale, the increasing urban population has brought massive pressure to resources and environment. It is the main direction of the low-carbon development of Bengbu city to advocate the concept of energy-saving development and accelerate the quality of urbanization construction. Although energy intensity has less impact on industrial carbon emissions than urbanization rate and the proportion of secondary industry, it is still an essential means to reduce industrial carbon emissions. Low-carbon development is still inseparable from the reduction of energy intensity, the adjustment of energy structure, and the optimization of industrial structure.

According to the grey prediction results, if the current population policy, economic growth policy and energy consumption structure are maintained, the industrial carbon emissions of Bengbu city will keep growing in the next decade. Although the growth rate is relatively slow, it will not reach its peak before 2030. This is also one of the characteristics of grey prediction model, which only shows a single trend. China’s industry occupies a large proportion of the national economy, and it is difficult to change the existing industrial structure and energy consumption mode in a short period, especially the dependence on coal resources. Such results are incredibly unfavorable to China and the global climate and environment. Through macro control and technological innovation, Bengbu’s industrial carbon emissions have been reduced in recent years, but there is still a big gap with China’s double carbon target, and continued efforts are needed in all aspects.

This study also has some limitations that can be further improved in future studies. On the one hand, this study only predicts carbon emissions under one scenario, and the simulation prediction under multiple scenarios can be carried out in the follow-up study using the scenario analysis method. On the other hand, since China is now vigorously promoting digital transformation, the subsequent research can be conducted from the perspective of the impact of digital economy on carbon emission reduction in industrial cities, and study how digital economy can empower carbon emission reduction in industrial cities to achieve low-carbon digital transformation in cities.

## Conclusions and recommendations

**Figure 4:**
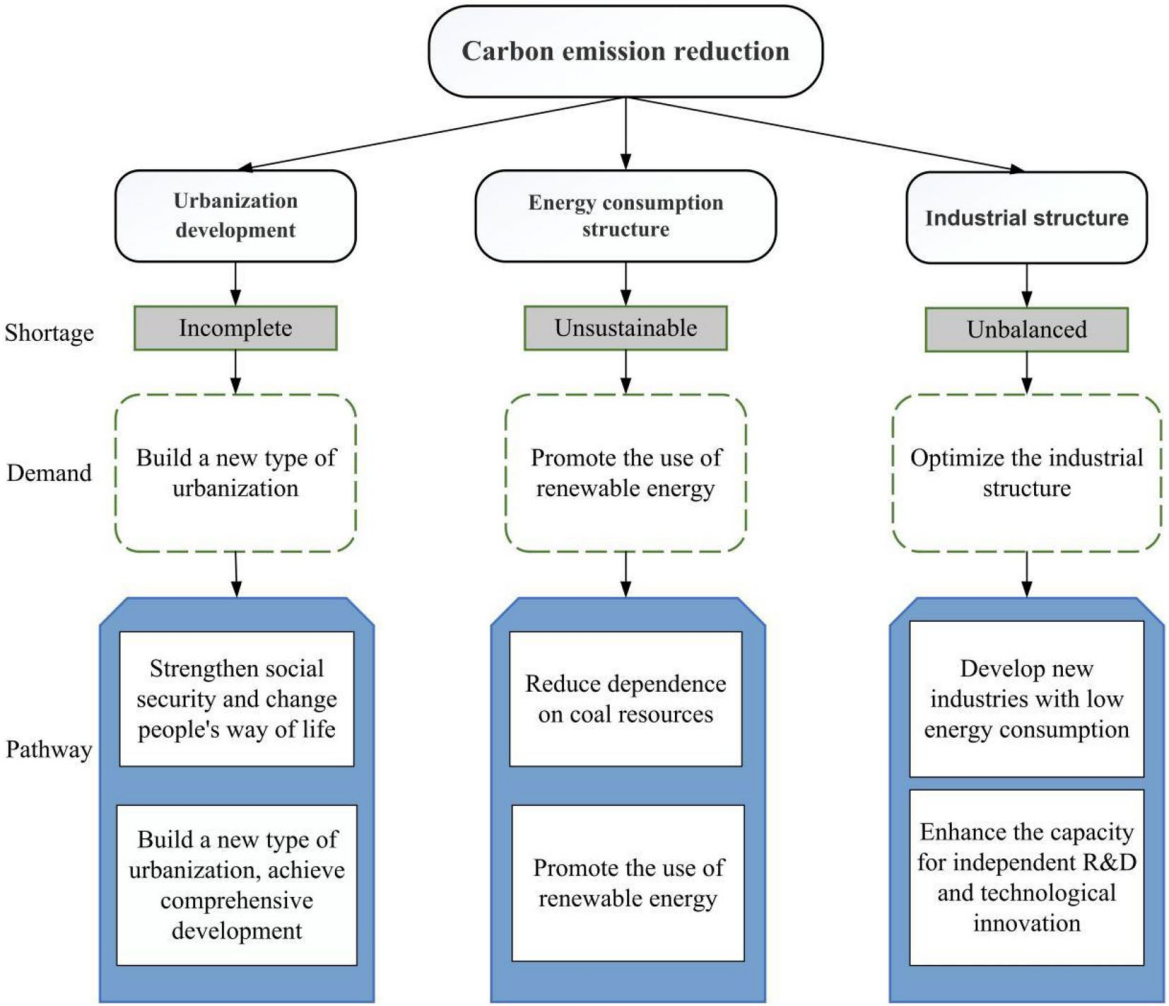
Shortages, demands and pathways of industrial cities to achieve carbon peak.

In this paper, we collected industrial energy-related data from 2011 to 2020 in Bengbu, an old industrial city, and analyzed the influencing factors and main resistance factors of industrial carbon emissions from five aspects: urbanization rate, industrial GDP, energy intensity, CO_2_ intensity, and the proportion of secondary industry, using CEI method and resistance model. Based on the industrial carbon emission data of Bengbu city from 2011 to 2020, the EGM (1,1) model and MATLAB 2017Ra software are used to predict the industrial carbon emission of Bengbu city from 2021 to 2030. The results show that industrial carbon emissions in Bengbu city will continue to increase in the next ten years, with a gradual growth rate, but will not reach a peak before 2030. Among the influencing factors, the urbanization rate has the most significant impact on industrial carbon emissions in Bengbu city, the secondary industry has a greater impact, and energy intensity has the least impact on industrial carbon emissions. Based on the main resistance factors of carbon emission, some recommendations for carbon emission reduction in old industrial cities are put forward, to ensure that can achieve carbon peak before 2030 (Fig. 4).

First, the pace of urbanization should be reasonably controlled to improve the quality of urbanization. According to the analysis results of CEI method, urbanization rate has the greatest impact on industrial carbon emissions. Therefore, in future urbanization construction, on the one hand, we should adhere to the new urbanization construction with people as the core and urban–rural integration, ecological livability and harmonious development as the main features. In the process of new urbanization construction, we should vigorously promote the construction of low-carbon cities and focus on urban green development. On the other hand, it should give full play to the reverse effect of carbon peaking, and guide the process of new urbanization by developing green low-carbon technologies and implementing energy-saving and emission reduction policies, to ultimately promote a higher level of coordinated development of the two.

Second, adjust the energy consumption structure and increase the use of green energy. Energy consumption in Bengbu is dominated by coal, and the proportion of clean energy consumption, such as natural gas, hydropower, nuclear power, and wind power, is relatively low. It can be seen that adjusting the energy consumption structure is also the key to achieving carbon peak and carbon neutrality in industrial cities. Therefore, in the future, the proportion of renewable energy consumption should be increased, and clean energy such as solar energy, wind energy, and hydrogen energy should be fully utilized. Increase the research of clean technology of coal, oil and natural gas, and gradually change renewable energy from supplementary energy to mainstream one. Optimize energy utilization technology and improve energy efficiency to reduce carbon dioxide intensity. Increase the development of renewable and clean energy, and reduce the share of coal in energy consumption.

Finally, optimize the industrial structure to help traditional industries upgrade. According to the analysis results of the resistance model, it is known that the proportion of secondary industry has a greater impact on industrial carbon emissions, and the energy intensity of secondary industry is much higher than that of primary and tertiary sectors. Therefore, industrial cities should actively optimize the industrial structure and vigorously develop advanced manufacturing, photovoltaic industry, and low-energy modern service industry. Increase the construction of comprehensive digital infrastructure such as artificial intelligence and industrial Internet to promote the automation, informatization, digitalization, and intelligent transformation of traditional industries. Furthermore, utilize digital technology to green and modernize industries and further promote the development of advanced industrial structures.

## Data Availability

The data used for this study could be made available on request with corresponding author.
